# Contribution to the taxonomy of *Garcinia* (Clusiaceae) in Africa, including two new species from Gabon and a key to the Lower Guinean species

**DOI:** 10.3897/phytokeys.17.3114

**Published:** 2012-10-24

**Authors:** Marc S.M. Sosef, Gilles Dauby

**Affiliations:** 1 Naturalis Biodiversity Center (section NHN), Wageningen University, Generaal Foulkesweg 37, 6703 BL Wageningen, The Netherlands; 2 Biosystematics group, Wageningen University, The Netherlands; 3 Laboratoire d’Evolution Biologique et Ecologie, Faculté des Sciences, Université Libre de Bruxelles, CP 160/12, Av. F.D. Roosevelt 50, 1050 Bruxelles, Belgique

**Keywords:** *Garcinia*, Clusiaceae, Africa, Lower Guinea, Gabon, taxonomy

## Abstract

*Garcinia* has some 260 species and is often regarded as a genus with a difficult taxonomy. No recent treatment is available for the botanically rich Lower Guinea phytogeographical region. This study aims at partly filling this gap. First, several taxonomic problems are solved. *Garcinia chromocarpa* is reduced to a variety of *Garcinia quadrifaria*. *Garcinia gnetoides* and *Garcinia granulata* are both synonyms of *Garcinia quadrifaria*. *Garcinia zenkeri* is a synonym of *Garcinia densivenia* and lectotypes are being designated for both names. *Garcinia brevipedicellata* is a synonym of *Garcinia afzelii*, as is *Garcinia antidysenterica* for which a lectotype is designated. Second, two new species endemic to Gabon are described: *Garcinia gabonensis* Sosef & Dauby and *Garcinia obliqua* Sosef & Dauby. Finally, an identification key to all species present in the Lower Guinea region is provided. A few remaining West African species names could not be placed with certainty, because the type material was lost or not traced yet. One is a Rutaceae while the remaining three are provisionally to be regarded as synonyms of *Garcinia smeathmannii*.

## Introduction

The genus *Garcinia* L. is part of the family Clusiaceae which, in its present circumscription (Stevens 2007), is subdivided into two sub-families: *Kielmeyeroideae* Engl., which includes the African genera *Mammea*, *Endodesmia*, *Lebrunia* and *Calophyllum*, and *Clusioideae* Engl. with the African genera *Allanblackia*, *Garcinia*, *Pentadesma* and *Symphonia*. All but one of these genera (*Lebrunia*) are present in the Lower Guinea phytogeographical region (western Central Africa; White 1979). Unfortunately, the delimitation of the family and its subdivision have not settled down yet (Stevens 2007, Sweeney 2008, APG III 2009).

Most authors now agree upon a broad concept of *Garcinia*, including the former genera *Rheedia* L. and *Ochrocarpos* Thouars, as well as the small genera *Pentaphalangium* Warb. and *Tripetalum* Schumann. One might also merge the genus *Allanblackia* with *Garcinia* (Sweeney 2008, Ruhfel et al. 2011), because the first seems phylogenetically nested within the latter, although the support for that is not very strong yet. Moreover, even when the nested position of *Allanblackia* is confirmed by future studies, regarding the large morphological differences between the latter two genera and hence the comparatively long length of the branch leading to *Allanblackia*, it will be preferable to accept *Garcinia* as a natural though paraphyletic genus (Sosef 1997, Brummitt and Sosef 1998).

Amongst the African genera, *Garcinia* is characterized by the dioecism of its species and hence its unisexual flowers (or at least functionally so, see also Dunthorn (2004) for a study of the situation in *Mammea*), the presence of a foveola at the base of the petiole (an excavation with an extension resembling a ligule), the peltate stigma, the ovary with a single apical ovule per locule and the berry-like fruit.

*Garcinia* contains approximately 260 species which are mainly confined to the tropics (Jones 1980, Stevens 2007). Over the centuries, these have been accommodated into many sections, which have been reduced to a total of 14 in an unpublished thesis by Jones (1980). Her view has been largely confirmed by recent molecular work (Sweeney 2008). In Africa, including Madagascar, representatives of six of these sections can be found.

In Lower Guinea (roughly comprising the forested regions in Cameroon, Equatorial Guinea, Gabon, the south-west of the Republic of the Congo, Cabinda (Angola) and the southwest of the Democratic Republic of the Congo; White 1979), and in fact in the whole of the African rain forest region, *Garcinia* spp. form an important component of the lower strata of dense lowland to submontane rain forests, where they often occur gregariously (Achoundong 1995, Guedje et al. 2002, Thomas et al. 2003, Sosef et al. 2004, Senterre 2005, Gonmadje et al. 2011, Dauby 2012). In Central Africa, some species are also well-known among local populations due to their various medicinal properties (Guedje and Fankap 2001, and see the PROTA website at http://www.protaafrica.org ).

The presence of high numbers of sympatric *Garcinia* species in almost every tropical region (Ashton 1988, Sweeney 2008) may well have led to a situation where the genus received comparatively little attention from taxonomists because of its supposed complexity. This has resulted in an unsatisfying situation where taxonomic treatments have often only been focussing on a particular region (in Floras for example) and as a result contain various contradictions and errors. This obviously further feeding the idea that *Garcinia* is a complex and ‘difficult’ genus. Plant collectors, but also foresters, ecologists, etc., are often satisfied when they have concluded that their specimen belongs to the genus *Garcinia* and when they try to identify their material down to an individual species, they are bound to make many errors due to the unresolved taxonomic backbone.

In tropical Africa, the Clusiaceae (often in their old concept of Guttiferae) have been treated for West Africa (Hutchinson et al. 1954), the Congo Basin region (Bamps 1970a), East Africa (Bamps et al. 1978), and the Zambesian region (Robson 1960). However, no treatment exists yet for the notoriously richest Lower Guinean region (Sosef 1996, Küper et al. 2004). Many West African species extend their range to the east into Lower Guinea while many East and Central African ones do so to the west. Combined with a comparatively high local endemism in Lower Guinea, possibly caused by the presence of Pleistocene rain forest refugia (Sosef 1994, 1996, Linder 2001), this area counts as a diversity hotspot for many angiosperm families (Beentje et al. 1994, Barthlott et al. 1996, Küper et al. 2004, Linder et al. 2005). Besides that, it is also the region where taxonomic views and treatments of ‘West’ and ‘East’ meet and not seldom appear to disagree. During the preparation of a treatment of the Clusiaceae for the revitalized Flore du Gabon (Sosef and Florence 2007), several of thesetaxonomic ‘problems’ within *Garcinia* were tackled, and it was decided to slightly broaden the scope of that study. The resulting present article clarifies the confusing taxonomy amongst several African members of the sections *Xanthochymus* (Roxb.) Pierre and *Tagmanthera* Pierre, describes two interesting new species from Gabon, provides an identification key to the *Garcinia*’s within thehigh diversity region of Lower Guinea, and hopes to draw attention to the need of more elaborate taxonomic studies in *Garcinia*.

## Materials and methods

The authors studied the available *Garcinia* material in BM, BR, BRLU, K, L, LBV, MO, P and WAG (herbarium abbreviations follow Index Herbariorum, http://sweetgum.nybg.org/ih ). Additional material was consulted through the JSTOR Plant Science website (http://plants.jstor.org ). In 2008 and 2009, during tree plot inventories and general collecting undertaken in Gabon, the second author collected numerous *Garcinia* specimens and made many useful field observations.

The conservation status of the two new species was assessed using the IUCN (2001) category criteria. Extent of occurrence and area of occupancy were estimated using Arcview 3.3 and Conservation assessment tools (Moat 2007).

## The *Garcinia quadrifaria* complex

### On the correct authors of the name *Garcinia quadrifaria*

The species now known as *Garcinia quadrifaria* was first described by Oliver (1868: 168) as *Xanthochymus ? quadrifarius*. It was transferred to *Garcinia* by Pierre (1883: 4) who seems to credit Baillon (1877: 404) for the name and also cites Oliver (1868: 168). Many sources (including IPNI, (http://www.ipni.org ), Tropicos (http://www.tropicos.org ), etc.) refer to *Garcinia quadrifaria* with Baill. or Baill. ex Pierre as the authors of this combination. Actually, Baillon (1877) only states that *Xanthochymus* should be united with *Garcinia*, and refers to Oliver (1868), but does not make any new combinations. Therefore, the correct author combination for *Garcinia quadrifaria* is (Oliv.) Pierre.

### On the distinction between *Garcinia quadrifaria* and related species

The species *Garcinia quadrifaria* belongs to the section *Xanthochymus*, characterized mainly by having staminal bundles with filaments only partly fused and globose anthers (Jones 1980). *Garcinia quadrifaria* shows several quite striking characters. Most obvious are the quadrangular and narrowly though distinctly winged twigs, the usually terminal inflorescence composed of a simple and short rachis set by 4 rows of overlapping scale-like bracts (somewhat reminiscent of an inflorescence of a *Gnetum* species), the 5-merous flowers, a fruit with a verrucose exocarp and white latex. It shares these features with the following, until recently still recognized, species: *Garcinia chromocarpa* Engl., *Garcinia gnetoides* Hutch. & Dalziel, *Garcinia granulata* Hutch. & Dalziel and *Garcinia le-testui* Pellegr. In the past, two more species names, *Garcinia parva* Spirlet and *Garcinia echirensis* Pellegr., have been treated as synonyms of *Garcinia chromocarpa* (Bamps, 1970a, 1970b).

After careful examination of the characters of *Garcinia quadrifaria* and *Garcinia chromocarpa*, we have come to the conclusion that the only distinction between the two is the minute puberulence on the bracts, pedicels and fruits of *Garcinia chromocarpa*,where those of *Garcinia quadrifaria* are glabrous. Although at first glance there might be a geographical distinction, *Garcinia chromocarpa* in the Congo Basin, west to Gabon and Cameroun (Bamps 1970c), and *Garcinia quadrifaria* in W.-Africa east to Cameroon and Gabon, this could not be upheld because of the presence of true *Garcinia quadrifaria* in eastern DR Congo (North Kivu: Léonard 2337; Kivu: Gutzwiller 1868) and true *Garcinia chromocarpa* in Ivory Coast (Breteler 6126, J.J. de Wilde & Leeuwenberg 3442). Also, when studying the literature (Engler 1908, Pellegrin 1959, Bamps 1970a, 1970b) one might conclude that *Garcinia chromocarpa* has 3-locular ovaries and 3-lobed stigmas, while *Garcinia quadrifaria* would have 2-locular ones bearing 2-lobed stigmas. Although indeed all true (glabrous) *Garcinia quadrifaria* specimens studied had 2-lobed stigmas, various true (puberulent) *Garcinia chromocarpa* ones with 2-lobed stigmas (D.W.Thomas 4874, Walters & Niangadouma 1264, White 767and others) were observed. Finally, from the literature it seems that flowers of *Garcinia chromocarpa* may have shorter pedicels than those of *Garcinia quadrifaria*. Again, after proper examination of all the material available this turns out to be incorrect.

We therefore conclude that since the differences between the two taxa are minimal, they cannot be upheld as different species. We do, however, want to distinguish them, and the level of variety seems most appropriate since there is no geographical separation. Because the name *Garcinia quadrifaria* has priority over *Garcinia chromocarpa*, this leads to the following new combination:

#### Garcinia quadrifaria chromocarpa

(Engl.) Sosef & Dauby comb. nov.

urn:lsid:ipni.org:names:77122646-1

Garcinia chromocarpa Engl., Bot. Jahrb. Syst. 40: 561 (1908). [Basionym]
Garcinia echirensis Pellegr., Bull. Soc. Bot. France 106 : 225 (1959). [Heterotypic synonym]
Garcinia parva Spirlet, Bull. Jard. Bot. État Bruxelles 29 : 326 (1959). [Heterotypic synonym]

Subsequently, we have studied the West-African species *Garcinia gnetoides* Hutch. & Dalziel. The type material at K, Chevalier 15157, consists of a plant carrying terminal inflorescences with a dense mass of many racemes composed of a short rachis with closely set bracts which are glabrous. In *Garcinia quadrifaria* each raceme normally appears solitary. An old note attached to the type already states it might well be a galled inflorescence, because a larva was observed inside. Hutchinson and Dalziel are well aware of its potentially diseased nature which shows from their remark in the Flora of West tropical Africa (Hutchinson and Dalziel 1927). However, in their more elaborate 1928 publication they do not mention the galled inflorescence. They cite two other specimens apart from the type (Chevalier 15620 and Vigne 222) and these have normal, solitary racemes. Besides that, they cite *Xanthochymus quadrifarius* A.Chev. non Oliv. as belonging here. So, they were aware of the fact that this plant had some relation to that species, transferred to *Garcinia* by Pierre in 1883 (see above). Their plants had no fruits. In the same 1928 publication they describe another new species, *Garcinia granulata*, citing a single specimen, Unwin & Smythe 58, that bears only fruits which are verrucose and glabrous.

After careful examination of all material at hand, we cannot conclude otherwise than that both *Garcinia gnetoides* and *Garcinia granulata* represent the same species known to us as *Garcinia quadrifaria* and thus are synonyms of the latter. The fact that the first two are in fact synonymous was already concluded by Hawthorne and Jongkind (2006).

Some sources cite the publication of the names *Garcinia gnetoides* and *Garcinia granulata* in Kew Bulletin (Hutchinson and Dalziel 1928) as the place of valid publication, and not that of one year earlier in the Flora of West tropical Africa (Hutchinson and Dalziel 1927). In the latter publication the authors indeed seem to indicate the names will be formally published later on by adding “ined.”, meaning *ineditus* (unpublished), behind “Kew Bull.”. However, this does not render their 1927 publication invalid. Article 34.1b of the Code (McNeil et al. 2006) does not apply, because they do accept the taxa.

So, in conclusion, the new situation is as follows:

#### Garcinia quadrifaria quadrifari

Garcinia gnetoides Hutch. & Dalziel, Fl. West trop. Afr. ed. 1, 1(1): 236 (1927), **syn. nov.** [Heterotypic synonym]
Garcinia granulata Hutch. & Dalziel, Fl. West trop. Afr. ed. 1, 1(1): 236 (1927), **syn. nov.** [Heterotypic synonym]

Finally, *Garcinia le-testui*, a rare species endemic to southern Cameroon and Gabon,seems sufficiently distinct from *Garcinia quadrifaria* being larger in most parts (notably wider wings on the twigs, larger leaves, longer pedicels, etc.). Most differences being related to size, it would not be surprising if *Garcinia le-testui* turns out to be a polyploid of *Garcinia quadrifaria*.

### On the status of *Garcinia densivenia* and *Garcinia zenkeri*

Two more species of the section *Xanthochymus* were described by Engler (1908): *Garcinia densivenia* and *Garcinia zenkeri*. The firstwas based on two collections from Cameroon: Zenker 2397 (with flowers) and Zenker 2547(with fruits). The angular twigs and coriaceous leaves are reminiscent of *Garcinia quadrifaria*. However, the fruit wall is smooth (not verrucose) and the inflorescence is both terminal and axillary and composed of bundles of very short racemes (up to 10 mm). Occasionally the inflorescence is composed of a branched raceme. In our view, this renders the taxon sufficiently distinct to recognize it at species level. To date, no lectotype has been chosen from among the two syntypes, and because the fruit character is the most striking distinction, the best choice would be Zenker 2547. With the duplicate at B lost, we propose to select the duplicate at G, which seems to have the finest fruits, as the lectotype.

*Garcinia zenkeri* was also based on two Zenkercollections from Cameroon: Zenker 1120 (with flowers) and Zenker 3247(with fruits). According to the African Plants Database (http://www.ville-ge.ch/musinfo/bd/cjb/africa ), *Garcinia zenkeri* would be a synonym of *Garcinia quadrifaria*. This view is probably based upon Pellegrin (1959), who only cites the first syntype. Studying material of both syntype collections (especially the first with numerous duplicates in various herbaria), we indeed confirm the presence of angular twigs and coriaceous leaves which point to *Garcinia quadrifaria*. However, we also observe the presence of inflorescences composed of bundles of short racemes, often axillarily positioned, just as in *Garcinia densivenia*. The only duplicate of Zenker 3247 (the syntype with fruits)available to us is sterile, and so we were unable to verify whether the fruit wall is smooth as in *Garcinia densivenia*. On the other hand, Engler l.c. does not mention a verrucose structure of the fruit wall, a feature we believe he would certainly not have missed. We therefore render it most likely that the fruit he observed had a smooth wall. In all, we conclude that *Garcinia zenkeri* and *Garcinia densivenia* are synonyms. Both being published in the same publication, we may choose one of the names as being the valid one, and we have picked *Garcinia densivenia* because the type material is better, showing all diagnostic features. *Garcinia zenkeri* also needs a lectotypification, for which we have taken Zenker 1120.Although all duplicates we have seen lack flowers, it is by far the most widely distributed collection. Since, again, the material at B was lost and that at G seems the best among the remaining duplicates available, we have chosen that to be the lectotype. The above leads to the following situation:

***Garcinia densivenia* Engl.**, Bot. Jahrb. Syst. 40: 563 (1908). ‒ LECTOTYPE (designated here): CAMEROON. Bipinde, Urwaltgebiet, 1903. Zenker 2547(G!, barcode G00018874; isotype BR!, GOET!, K!, M!, P!, WAG!).

Heterotypic synonym:

*Garcinia zenkeri* Engl., Bot. Jahrb. Syst. 40: 566 (1908), **syn. nov.** ‒ LECTOTYPE (designated here): CAMEROON: Bipinde, Urwaltgebiet, 1896, Zenker 1120(G!, barcode G00018871; isotype BM!, GOET!, K!, M!, P!, S!, WAG!).

The material now identified as *Garcinia densivenia*, shows a remarkable variation in the distinctiveness of the tertiary venation. In the type as well as the paratype collection this venation is indeed, as the name indicates, strikingly dense and prominent. However, in most of the remaining material we observed a large continuous variation towards leaves where the tertiary venation was even hardly visible. Because otherwise, the material is quite uniform, we assume this character to be highly variable within the species, and possibly also depending on the way in which the material was treated and dried after collecting.

A second remarkable variation was observed in the shape of the fruits. These can be subglobose to distinctly 5-lobed and ‘pumpkin-like’. The lobed feature was even already mentioned by Engler in his protologue: “Baccae ….. leviter 5-lobae…..” and “schwach 5 lappigen Früchte…..”. However, again, we found no other characters to correlate with this feature. Moreover, the label of Bos 3639 specifically mentions that the shape of the fruits he collected are “globose to shallowly 5-lobed and resembling a pumpkin”. We thus assume that these observations also illustrate within-species variation, and might be related to the number of seeds that develop within a single fruit.

### The *Garcinia afzelii* complex

The section *Tragmanthera* is characterized by 4-merous flowers and staminal bundles that are completely fused carrying a row of ellipsoid anthers at their tip (Jones 1980). Four of its species, *Garcinia afzelii* Engl., *Garcinia brevipedicellata* (Bak.f.) Hutch. & Dalziel, *Garcinia lujae* De Wild. and *Garcinia mannii* Oliv., are closely related because they share a unique feature: the presence of anthers with locellate (septate) thecae. After studying the material, we noticed it is fairly easy to split it into two groups based on a leaf venation character: 1) *Garcinia afzelii* and *Garcinia brevipedicellata* having leaves with the lateral veins (3-)4-11 mm apart and gradually but distinctly curving up towards the margin and eventually running almost parallel to it, and 2) *Garcinia lujae* and *Garcinia mannii* with dense lateral veins, only 1-2(-3) mm apart, that run almost straight to the margin, or curving up just before it, where they join up in a blunt angle with an intramarginal vein running just inside of (at 0.5-1 mm) the actual margin.

Bamps (1970b) already noticed that the latter two species are very closely related. The only remaining differences we could find is the colour of the petals (red to deep orange or sometimes yellow in *Garcinia mannii* and always yellow in *Garcinia lujae*) and the length of the staminal bundles (as long as the pistillode in *Garcinia mannii* and clearly overtopping the pistillode in *Garcinia lujae*). Although these distinctions are weak, there is no geographical overlap between the two taxa, *Garcinia lujae* in the Democratic Republic of the Congo and *Garcinia mannii* from southern Nigeria to Gabon, which made us decide to uphold them at species level, but further investigations are needed.

However, we could not find any clear difference between the remaining two species, *Garcinia afzelii* and *Garcinia brevipedicellata*. Hutchinson et al. (1954) give the density of the lateral veins (more dense in *Garcinia afzelii*) as the only difference, but we have clearly observed this feature to be variable, even within a single specimen. Also, the name of *Garcinia brevipedicellata* suggests it has short pedicels (although this taxon was originally described as a variety of *Garcinia mannii* and so this would be a difference with that taxon), but again, those in *Garcinia afzelii* are quite variable and we observed a continuous variation. Finally, there might be a difference in flower colour, since the petals of *Garcinia afzellii* are reported to be yellow to pale green, while label data indicates the flowers of *Garcinia brevipedicellata* are “yellow with a red centre”. The latter observation is indeed correct, because the petals are yellow and the pistillode or ovary has an orange to red colour. Both species have staminal bundles that are longer than the ovary. So, none of the presumed diagnostic characters can be confirmed and we conclude that both names refer to the same species. Since *Garcinia afzelii* is the older one, that is the accepted name for the taxon.

Finally, the status of the name *Garcinia antidysenterica* A.Chev. is unclear. At present, it is regarded as a synonym of *Garcinia afzelii* (Hutchinson et al. 1954, Pellegrin 1959, African Plants Database at http://www.ville-ge.ch/musinfo/bd/cjb/africa ). The protologue text provides information on three collecting localities referring to Chevalier’s own specimens: “entre Nze et Danané”, “entre le Morénou et l’Indénie” and “bassin du moyen Comoé”. No holotype is designated, but the protologue is accompanied by a photo of one of the sheets: Chevalier 21200, collected at “Danané”, and so this seems to be the most logical choice for the lectotype. However, this collection (duplicates at P and K) turns out to be *Garcinia epunctata* Stapf, as was already discovered by Bamps (1969: 364). While the protologue clearly states that the anthers have locellate thecae, those of *Garcinia epunctata* and hence of Chevalier 21200 are not. Therefore, choosing the latter collection as the holotype should be avoided, following Article 9.17 of the Code (McNeil et al. 2006; lectotype in serious conflict with the protologue while other elements are available). Chevalier (1920) provides a list of specimens which according to him belong to *Garcinia antidysenterica*. The only other collection that carries one of the remaining two localities, “vallée du Moyen-Comoé”, is Chevalier 22571. Therefore, this seems the most obvious choice for the lectotype. It has at least three duplicates (BR, K (2x) and P) and the specimen at P should be regarded as the most original material. At least the BR and K duplicate carry an original label with the name *Garcinia antidysenterica* A.Chev. (The P material was seen at an early stage of the project, but due to the closure of the Paris herbarium the exact label data could not be checked.)

The above now leads to the following situation:

***Garcinia afzelii* Engl.**, Bot. Jahrb. Syst. 40: 570 (1908).

Heterotypic synonyms:

*Garcinia antidysenterica* A.Chev., Vég. ut. Afr. trop. franc. 6: 445, fig. 52 (1911). ‒ LECTOTYPE (designated here): IVORY COAST: vallée du Moyen-Comoé, entre Yabrouakrou et Tingouéla, 13 décembre 1909, Chevalier 22571 (P!; isotype BR!, K!).

*Garcinia mannii* Oliv. var. *brevipedicellata* Bak.f., in Rendle *et al*., Cat. pl. Oban* *: 8 (1913). – *Garcinia brevipedicellata* (Bak.f.) Hutch. & Dalziel, Fl. West trop. Afr., ed. 1, 1(1): 237 (1927), **syn. nov.**

Within the section *Tragmanthera* some taxonomic questions remain to be solved, for example the distinction between the non-locellate species such as *Garcinia epunctata* Stapf and *Garcinia preussii* Engl., and the status of several other names now regarded as their synonyms. For now, we maintain the present status quo, just signalizing the need for a more in-depth study.

### Two new endemic *Garcinia* species from Gabon

During the preparation of the Clusiaceae treatment for Flore du Gabon, material belonging to two new species turned up. Both are endemic to this country that has a plant endemism rate of ca. 11% (Sosef et al. 2006). Gabon is notoriously rich in species (see above), especially its lowland rain forest is reputedly the most species-rich in tropical Africa (Breteler 1990, Linder et al. 2005, Reitsma 1988) and novelties are still regularly discovered (Bissiengou and Sosef 2008, Ntore et al. 2010, Walters et al. 2011).

#### Garcinia obliqua

Sosef & Dauby

urn:lsid:ipni.org:names:77122647-1

http://species-id.net/wiki/Garcinia_obliqua

Fig. 1

##### Diagnosis.

Similar to *Garcinia smeathmannii* but leaves with 6‒9 pairs of lateral veins, tertiary venation only slightly distinct above, petiole almost smooth, and fruits asymmetric with a coriaceous and ribbed skin.

##### Type. 

GABON: Ogooué-Ivindo, near Djidji, 5‒10 km West of Koumémayong, 0°15'N, 11°50'E (DMS), 25-4-1988, Breteler 8993 (holotype WAG!; isotype BR!, K!, LBV!, MO!, P!).

##### Description.

Dioecious tree; bole up to 35 cm dbh; twigs round to slightly angular in cross section; latex transparent or yellow. *Leaves* opposite; petiole (0.8‒)1‒1.5(‒2) cm long, smooth or slightly transversely wrinkled, with a distinct foveola of up to 3 mm long ; blade elliptic to lanceolate, (8‒)9‒17(‒18) × 2.5‒6 cm, attenuate at base, acuminate at apex, glabrous; lateral veins 6‒9 pairs, distinct below, slightly distinct above, disappearing towards the margin; tertiary venation visible to distinct below, slightly distinct above; resin canals visible on the lower surface, black and subparallel to the midrib. *Inflorescence* axillary, with fascicles of flowers on swellings in the axils of twigs. *Flowers* 4-merous, unisexual; pedicel 3‒6 mm; external sepals about 2.5 mm long, internal ones about 3 mm, greenish; petals suborbicular, about 4 mm long. *Male flower*: staminal bundles tree-like, with 10‒19 irregularly positioned stamens per bundle, filaments free at the apex, anthers small, subglobose; pistillode present. *Female flower* not observed. *Fruit* on a 12 mm long pedicel (only 1 fruit with pedicel observed), asymmetric, oblique, probably because of aborted ovules, circular to broadly ovate in cross section, 3‒3.5 × 1.8‒2 cm; **e**xocarp strongly furrowed and coriaceous and green with blue reflection in dry condition. *Seed* 1, obliquely ellipsoid, 2‒3 × 1.5‒2 cm, black.

**Figure 1.**
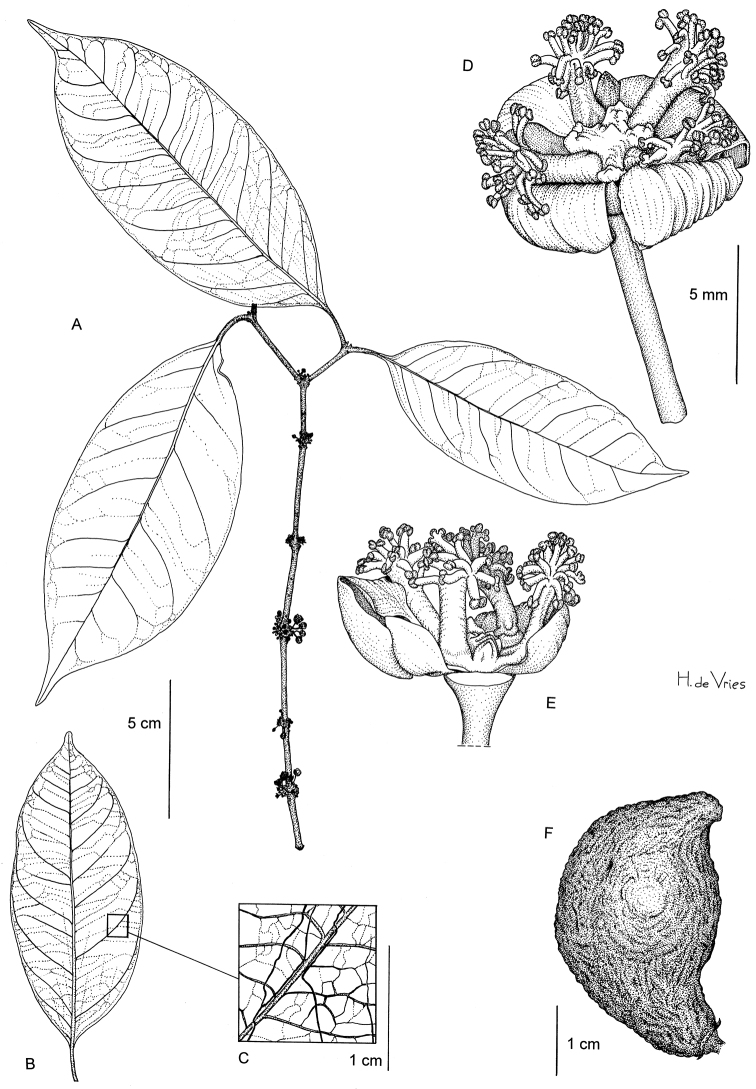
*Garcinia obliqua*: **A** Flowering twig **B** Leaf from below **C** Idem, detail **D** Male flower **E** Idem, 1 sepal and 2 petals removed **F** Fruit. (A: Breteler et al. 8993 **B, C, F**: Dauby et al. 1570; **D, E**: Breteler et al. 8738). Drawing by Hans de Vries, NCB Naturalis (section NHN) ^©^.

##### Distribution.

Endemic to central Gabon, known only from the Ogooué-Ivindo and Ngounié provinces (see Figure 3a).

##### Habitat and ecology.

Primary terra firme rainforest; at ca. 450‒800 m altitude. Flowering in April, fruits observed in February.

##### Conservation status.

*Garcinia obliqua* is currently known from nine collections and six locations. Estimates of its extent of occurrence and area of occupancy are respectively ca. 9488 km^2^ and 80 km^2^. One collection (Dauby et al. 1650) corresponded to a dead individual along a forestry road and all locations are currently found within logging concessions. Hence, we assume the extent of occurrence, area of occupancy, quality of habitat and number of sub-populations will decrease in the near future. We therefore assign a preliminary status of Vulnerable (Vu B1ab(i,ii,iii,iv,v)+B2ab(i,ii,iii,iv,v)).

##### Notes.

The shape of the staminal bundles and the anthers, as well as the distinct foveola point to a relationship with *Garcinia smeathmannii* and *Garcinia ovalifolia*, both belonging to the section *Rheediopsis* Pierre (Jones, 1980). On the other hand, preliminary molecular data obtained by the second author suggest that *Garcinia obliqua* is not related to these species. Therefore, the results of an upcoming molecular study are awaited before a firm statement about the sectional position can be made.

##### Additional specimens examined

(all from Gabon). CFAD de Rimbunan Hijau, au Sud-Ouest du Parc National de la Lopé, 0.64°S, 11.16°E (DD), 2/2/2009 (fr.), Dauby et al. 1570 (LBV, MO, BRLU). CFAD de Rimbunan Hijau, au Sud-Ouest du Parc National de la Lopé, 0.7°S, 11.23°E (DD), 27/2/2009 (fr.), Dauby et al. 1643 (BRLU). CFAD de Rimbunan Hijau, au Sud-Ouest du Parc National de la Lopé, 0.7°S, 11.23°E (DD), 28/2/2009 (ster.), Dauby et al. 1650 (BRLU). near Djidji, 5‒10 km W. of Koumémayong, 0°15'N, 11°50'E (DMS), 15/4/1988 (fl.), Breteler et al. 8738 (WAG). Est du Parc National de Waka, à plus ou moins 5 km au Sud de la rivière Mayi, 1.23°S, 11.28°E (DD), 4/6/2008 (ster.), Dauby et al. 677 (BRLU). Est du Parc National de Waka, à plus ou moins 5 km au Sud de la rivière Mayi, 1.23°S, 11.28°E (DD), 4/6/2008 (ster.), Dauby et al. 666 (BRLU). Bouvala hills, 1.62°S, 11.75°E (DD), 8/10/2007 (ster.), MBG transect (Leal et al.) 1105 (BRLU). Bouvala hills, 1.63°S, 11.78°E (DD), 12/10/2007 (ster.), MBG transect (Leal et al.) 1106 (BRLU). Village Eghuba, nord-ouest du Parc National de Waka, 1.03°S, 11.14°E (DD), 12/5/2008 (ster.), Ngombou Mamadou & Ndjombe 226 (LBV, MO).

#### Garcinia gabonensis

Sosef & Dauby

urn:lsid:ipni.org:names:77122648-1

http://species-id.net/wiki/Garcinia_gabonensis

Fig. 2

##### Diagnosis.

Similar to *Garcinia kola*, but leaves with lateral veins towards the margin clearly connected in distinct loops and united into an intramarginal vein that runs at (1‒)2‒3 mm from the margin, free stamens and a well-developed, longitudinally ribbed pistillode.

##### Type.

GABON: Ngounié, c. 36 km Mouila to Yeno, 1°45'S, 11°20'E, 19-9-1986, Breteler 7782 (holotype WAG!; isotype BR!, K!, LBV!, MO!, P!, PRE).

##### Description.

Dioecious shrub or small tree, up to 4 m high; latex transparent to greenish; branches circular in cross section, fissured, often reddish when dry; twigs flattened on cross section, smooth. *Leaves* opposite; petiole (4‒)5‒10(‒12) mm, smooth, slightly canaliculated above, with indistinct foveola of about 1 mm long; blade generally oblanceolate, sometimes elliptic or rarely ovate, (7‒)8‒15(–16) × (2–)2.5–5(‒6) cm, pointed at base, caudate-acuminate at apex, coriaceous to papery, glabrous; midrib prominent below, canaliculate above, lateral veins 7‒13 pairs, visible on both surfaces**,** towards the margin clearly connected in distinct loops and united into an intramarginal vein that runs at (1‒)2‒3 mm from the margin, tertiary veins laxly reticulate, indistinct; resin ducts normally indistinct except in young leaves, subparallel to the midrib. *Inflorescence* axillary, of few-flowered fascicles; bracts many, small (<1 mm long). *Flower* 4-merous, unisexual; pedicel slender, 2(‒3) mm; sepals obovate, two external ones about 2 mm long, two internal ones about 4 mm long, greenish to yellowish; petals obovate, about 4 mm long, yellowish or greenish to white. *Male flower*: stamens 8‒14, free, inserted in a ring around the pistillode, filament broadened and flat, white, anthers ellipsoid, strongly curved; pistillode broadly triangular-obovoid, longitudinally ribbed, stylode simple and slender, 1‒2 mm long. *Female flower*:disc annular, flattened and pressed against the ovary; ovary globose, about 3 mm in diameter; stigma peltate, lobed, 2 mm wide. *Fruit* ovoid to subglobose, 5‒11 mm in diameter, greenish, smooth, with persistent sepals at base.

**Figure 2.**
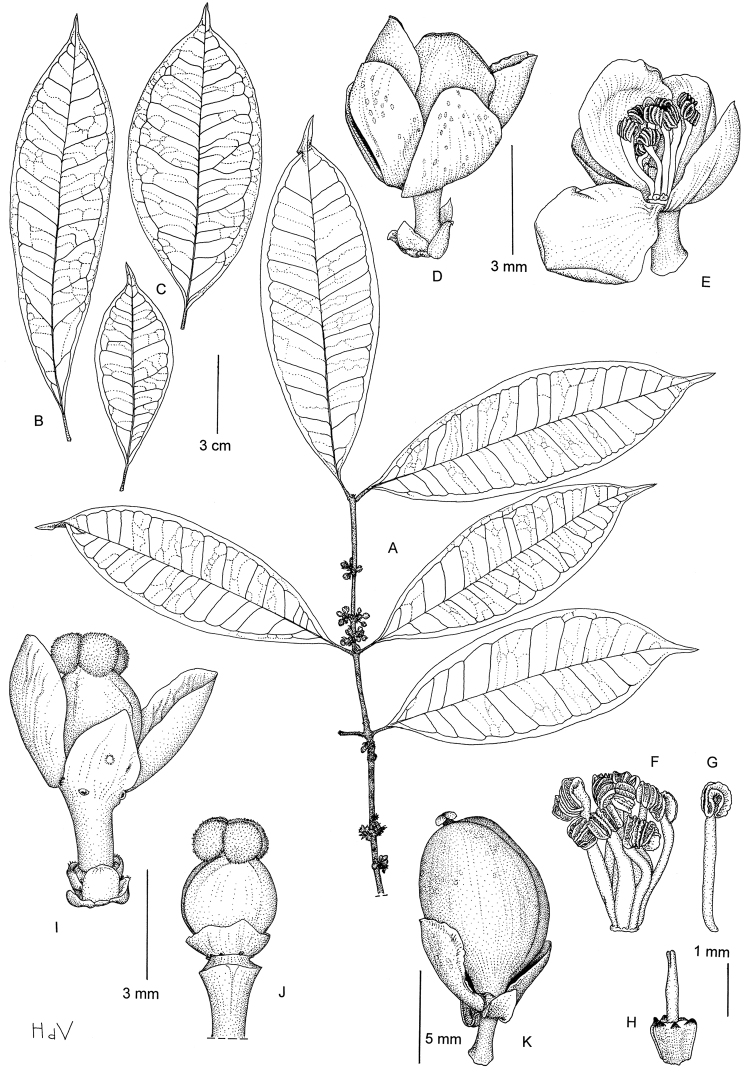
*Garcinia gabonensis*: **A** Flowering twig **B, C** Leaves, showing the variation **D** Male flower **E** Idem, open and three petals removed **F** Androecium **G** Stamen **H** Pistillode **I** Female flower **J** Gynoecium and disk **K** Fruit. (**A, D–H**: Leeuwenberg & Persoon 13683; **B, K**: Arends et al. 510; **C, I, J**: Wieringa et al. 4546). Drawing by Hans de Vries, NCB Naturalis (section NHN) ^©^.

##### Distribution.

**Figure 3.**
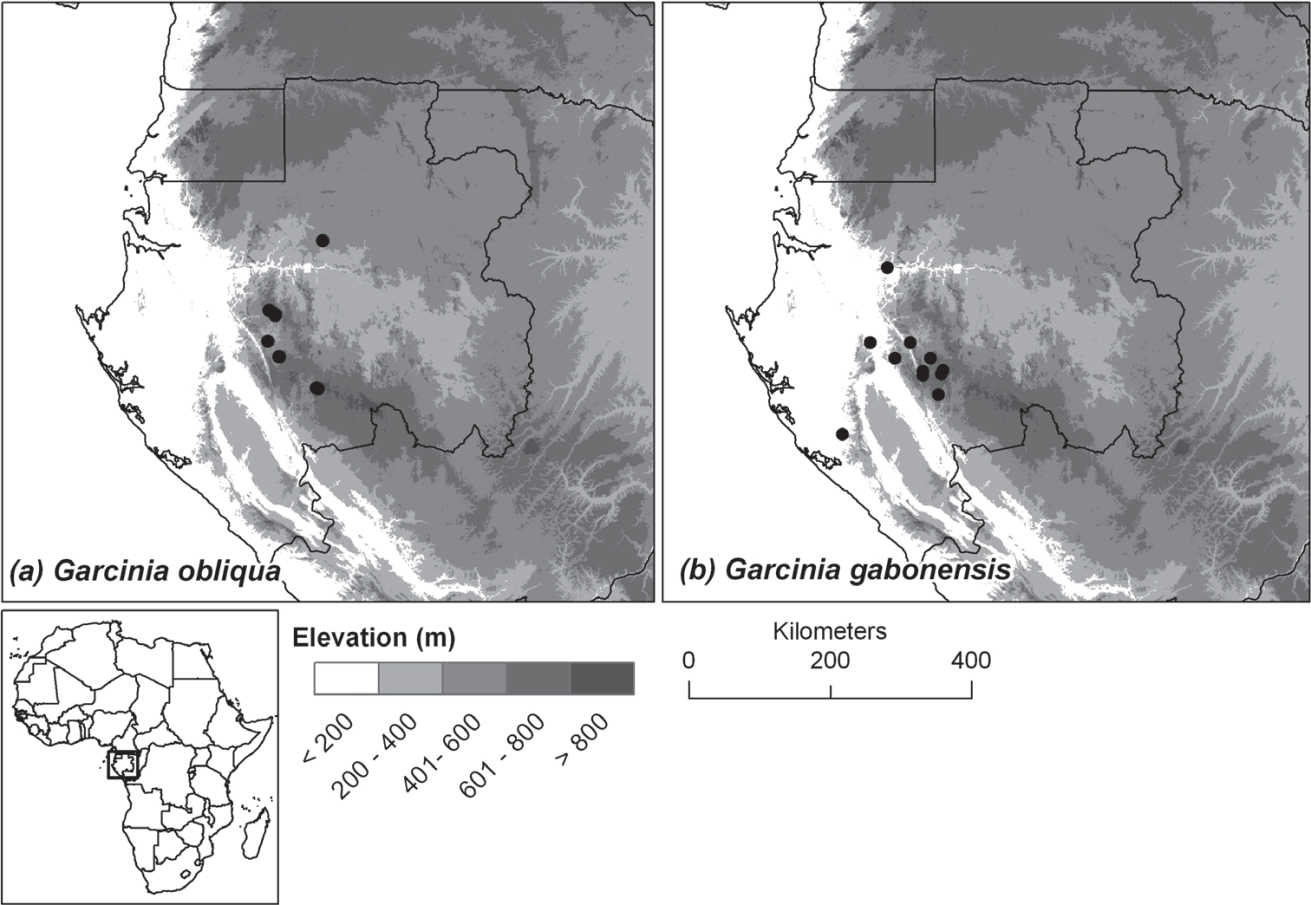
Distribution of the two new species endemic to Gabon: (**a**) *Garcinia obliqua*, (**b**) *Garcinia gabonensis*.

Endemic to southern and central Gabon, in the provinces of Moyen-Ogooué, Ngounié and Ogooué-Maritime (see Figure 3b).

##### Habitat and ecology.

Primary or late secondary *terra firme* rain forest, along rivers or on ridges; at ca. 150‒850 m altitude. Flowering in September to November, fruiting in September, November, December and February.

##### Conservation status.

Currently, *Garcinia gabonensis* is known from eleven collections and nine locations. Estimates of the extent of occurrence and the area of occupancy are respectively ca. 16 000 km^2^ and 109 km^2^. Since nine of the eleven collections are within logging concessions or along main roads, we consider that continuing decline in the extent of occurrence, area of occupancy, quality of habitat and number of sub-populations has occurred or will occur in the near future. We therefore assign a preliminary status of Vulnerable (VU B2ab(i,ii,iii,iv)).

##### Notes.

For now, it remains unclear as to which section this species belongs. Most striking feature are the free stamens. According to the elaborate work of Jones (1980), this is characteristic for only two sections: sect. *Teracentrum* Pierre and sect. *Rheedia* sensu Jones. Species belonging to the first, however, have their stamens inserted across a central mass and lack a pistillode. Those belonging to the second are known to occur, until now, only in Central and South America and on Madagascar…. Morphologically though, *Garcinia gabonensis* seems very similar to other species in sect. *Rheedia*, and this might be the first continental African representative of that section. A molecular study to further investigate this will be performed soon.

##### Additional specimens examined

(all from Gabon). SE of Sindara, km 12 from Camp Chantier Waka to Ngounié River, 1°14'S, 10°51'E (DMS), 26/9/1985 (fl.), Leeuwenberg & Persoon 13683(BR, K, WAG). Moukabo, about 37 km E of Mouila, on the road to Yeno, 1°40'S, 11°20'E (DMS), 27/11/1984 (fl.), Arends et al. 484 (WAG). about 40 km E of Mouila, on the road to Yeno, 1°40'S, 11°20'E (DMS), 28/11/1984 (fr.), Arends et al. 510(BR, LBV, WAG). Massif du Chaillu, old secondary forest partly primary, near Mouyanama, about 27 km. E. of Mimongo, 1°39'S, 11°46'E (DMS), 25/11/1983 (fl.), Louis et al. 854 (K, WAG). Fougamou, 7 km on forestry road following Bendolo river, 1°12.1'S, 10°32.2'E (DDM), 26/10/1994 (fl.), Wieringa et al. 2916 (LBV, WAG). 10 km on the road Ikobey to Bakongue, Eghaba Mountain, 1°2.0'S, 10°2.6'E (DDM), 28/11/2001 (fl., fr.), Wieringa et al. 4473 (WAG). 5‒15 km NNW of Ndjolé, 0°5'S, 10°45'N (DMS), 13/11/1991 (fl.), Breteler 10445 (LBV, WAG). 13 km on the road Eteké to Ovala, Nyongué, 1°26.1'S, 11°26.1'E (DDM), 8/11/1994 (fl.), Wieringa et al. 3096(WAG). 60 km on the road Mouila to Yeno, 1°41.85'S, 11°23.96'E (DDM), 3/12/2001 (fr.), Wieringa et al. 4546 (LBV, WAG). Doudou mountains, about 60 km along exploitation track in WNW direction from Doussala, 2°12'S, 10°11'E (DMS), 27/11/1986 (fl.), Wilde J.J. de et al. 8984 (K, LBV, WAG). Massif du Chaillu, near Guédévé village about 40 km N of Lébamba, 1°55'S, 11°25'E (DMS), 30/11/1983 (fr.), Louis et al. 1056 (K, WAG). Est du Parc National de Waka, à environ 5 km au Sud de la rivière Mayi, 1°23'S, 11°3'E (DMS), 21/2/ 2008 (fr.), Dauby et al. 735 (LBV, MO, BRLU).

### Key to the Lower Guinean species of *Garcinia*

For most of the continental tropical African regions, an identification key to the species of *Garcinia* exists: Hutchinson et al. (1954) for West Africa, Bamps (1970a) for Central Africa, Bamps et al. (1978) for East Africa, and Robson (1961) for the Zambesian region. The lack of such a key for the notoriously species-rich Lower Guinean region has hindered proper identification of specimens; a shortcoming we hope to overcome by providing the key below.

**Table:** 

1	Inflorescence very large, central axis often over 50 cm and up to 180 cm long, with several very long and unbranched ramifications of similar lengths, carrying distantly spaced clusters of small white sessile flowers; leaf blade (14‒)25‒57 cm long, shiny	*Garcinia lucida* Vesque
–	Inflorescence much smaller; flowers at least shortly pedicellate; leaf blade normally smaller, up to 28(‒35) cm long, shiny or not	2
2	Twigs angular, slightly or sometimes distinctly winged; petioles transversely wrinkled; latex white; flowers 5-merous, in compact racemes with a tetragonous rachis and imbricate bracts; fruit smooth or verrucose	3
–	Twigs rounded to angular; petioles smooth to transversely wrinkled; latex yellow or transparent; flowers 4-merous, in fascicles, cymes or solitary; fruit smooth	6
3	Twigs narrowly winged; pedicel up to 1.5 cm long (up to 2 cm in fruit); leaf blade 5‒21 × 2‒9.5 cm	4
–	Twigs strongly winged, wings 3‒5 mm wide; pedicel 3‒5.5 cm long; leaf blade (14‒)18‒41 × (4.5‒)6‒15.5 cm	*Garcinia le-testui* Pellegr.
4	Ovary and fruit verrucose; inflorescence almost strictly terminal, 1(‒3) racemes together of 1.5‒3 cm long	5
–	Ovary and fruit smooth; inflorescence terminal and axillary, often with several racemes together or racemes branched, these 2‒10 mm long	*Garcinia densivenia* Engl.
5	Bracts, pedicels and fruit minutely puberulous	*Garcinia quadrifaria* (Oliv.) Pierre var. *chomocarpa* (Engl.) Sosef & Dauby
–	*Garcinia quadrifaria* (Oliv.) Pierre var. *quadrifaria*
6	Filaments fused, at least at base; leaf blade without intramarginal vein or with one that runs just inside (at 0.5‒1 mm) of the margin	7
–	Filaments entirely free; leaf blade with a distinct intramarginal vein running at (1‒)2‒3 mm from the margin	*Garcinia gabonensis* Sosef & Dauby
7	Staminal bundles with filaments partly free, at least at the top, and globose or ovoid anthers; leaf blade coriaceous	8
–	Staminal bundles with filaments completely fused and ellipsoid to oblong, curved anthers; leaf blade papyraceous to coriaceous	13
8	Leaf blade with distinct to very striking reticulations, green to brown in dry condition; sepals smooth to rugose; anthers 3‒20 per staminal bundle	9
–	Leaf blade with indistinct reticulations, brown-red when dry; sepals finely papillose; anthers very numerous	*Garcinia conrauana* Engl.
9	Pedicels and sepals glabrous; sepals smooth or slightly rugose; inflorescence axillary	10
–	Pedicels and sepals puberulous; sepals distinctly rugose; inflorescence terminal	*Garcinia kola* Heckel
10	Stamens 3‒10 per bundle; leaf blade with 15‒20(‒25) pairs of lateral veins; petiole distinctly transversely wrinkled; fruit symmetric	11
–	Stamens 11‒19 per bundle; leaf blade with 6‒9 pairs of lateral veins; petiole smooth to slightly transversely wrinkled; fruit oblique	*Garcinia obliqua* Sosef & Dauby
11	Leaves distinctly petiolate (petiole >4 mm long), with cuneate to rounded or seldom subcordate base	12
–	Leaves subsessile, with cordate and sometimes amplexicaulous base	*Garcinia staudtii* Engl.
12	edicel 1.5‒6(‒10) mm long; staminal bundles in male flower with 3(‒4) stamens; petiole 1‒2 mm thick; leaf blade 3‒15 × 0.5‒6 cm, usually long acuminate but the very top rounded	*Garcinia ovalifolia* Oliv.
–	Pedicel (10‒)15‒45 mm long; staminal bundles in male flower with (5‒)6‒10 stamens; petiole 2‒4 mm thick; leaf blade 8‒28(‒35) × 3,5‒12(‒17) cm, rounded to tapering or acuminate towards the top, the very top usually acute	*Garcinia smeathmannii* (Planch. & Triana) Oliv.
13	Leaf blade with lateral veins making an angle of (45‒)60‒80° with the midrib	14
–	Leaf blade with lateral veins making an angle of 30‒45° with the midrib	*Garcinia buchananii* Baker
14	Leaf blade opaque or with continuous translucent resin canals	15
–	Leaf blade with translucent resin canals composed of dots and short lines	*Garcinia punctata* Oliv.
15	Leaf blade with a distinct acumen to gradually acuminate; with main lateral veins (3‒)4‒11 mm apart, the intermediate ones often clearly not reaching the margin; in dry condition resin canals running parallel to the lateral veins indistinct or invisible; petals white to yellow or yellowish green, not sticky; staminal bundles longer than the pistillode, anthers with septate or non-septate thecae; mature fruit yellow to orange	16
–	Leaf blade with a distinct acumen; main lateral veins 1‒2(‒3) mm apart, because the intermediate ones are almost equally strong and often reach the margin; in dry condition those resin canals running parallel to the lateral veins often distinct and prominent; petals red or orange-red or sometimes yellow, often sticky; staminal bundles as long as the pistillode, anthers with septate thecae; mature fruit orange to purplish red	*Garcinia mannii* Oliv.
16	Leaf blade with lateral veins almost straight, curved just before the margin to be united with an intramarginal vein; petals white to pale yellow or yellowish green; thecae not septate	17
–	Leaf blade with lateral veins gradually and distinctly curved up towards the margin and finally subparallel to it; petals yellow to pale green; thecae septate	*Garcinia afzelii* Engl.
17	Flowers and fruits on a 1‒4 mm long pedicel	*Garcinia epunctata* Stapf
–	Flowers and fruits on a 7‒18 mm long pedicel	*Garcinia preussii* Engl.

### Remaining insufficiently known species

#### Garcinia arbuscula

Engl.

http://species-id.net/wiki/Garcinia_arbuscula

Garcinia arbuscula - Protologue: Bot. Jahrb. Syst. 55: 391 (1919).

##### Syntypes:

CAMEROON. Mfongu, am Muti-abhang, 1700‒1900 m alt., Ledermann 5863 & 5943. Not located, probably lost at B, no duplicates traced yet.

The protologue states there are 20‒30 stamens in 4 bundles (so some 5‒8(‒9) per bundle), fused until halfway, and leaves similar to *Garcinia ovalifolia*, but with less pronounced veins and a cuneate base, on 1‒1.5 cm long petioles. Flowers are positioned in glomerules on the twigs, below the leaves. This description fits that of *Garcinia smeathmannii* and we momentarily place this name under that species.

#### Garcinia danckelmanniana

Engl.

http://species-id.net/wiki/Garcinia_danckelmanniana

Garcinia danckelmanniana - Protologue: Bot. Jahrb. Syst. 55: 394 (1919).

##### Syntypes:

CAMEROON. Genderogebirge, Tschape pass, 1420 m alt., Ledermann 2671 & 2750. Not located, probably lost at B, no duplicates traced yet, but a sketch of Ledermann 2750 is present at BM.

The protologue states there are 30‒40 stamens in 4 bundles, fused almost to the top, a distinct foveola, leaves with 12‒15 lateral veins and flowers in many-flowered bundles on the nodes. The sketch at BM shows large leaves (12–19 × 3.5–8 cm) with an acute apex and flowers on pedicels of 1.5–2 cm. All this fits *Garcinia smeathmannii* best, and for the moment we regard it as a synonym of that species.

#### Garcinia laurifolia

Hutch. & Dalziel

http://species-id.net/wiki/Garcinia_laurifolia

Garcinia laurifolia - Protologue: Fl. W. trop. Afr. 1: 236 (1927).

##### Type:

SIERRA LEONE. Scott-Elliot 4806.

The type collection was located through the JSTOR Plant Sciences website (http://plants.jstor.org ) at BM. It was identified as a Rutaceae belonging to the genus *Teclea*. It shows a twig with alternate leaves and a single young fruit.

#### Garcinia tschapensis

Engl.

http://species-id.net/wiki/Garcinia_tschapensis

Garcinia tschapensis - Protologue: Bot. Jahrb. Syst. 55: 393 (1919).

##### Type:

CAMEROON. Genderogebirge, Tschape pass, 1430 m alt., Ledermann 2771. Not located, probably lost at B, no duplicates traced yet, but a sketch of Ledermann 2771 is present at BM.

The protologue states the material concerns a fairly large tree (18‒22 m), with twigs soon rounded, rugose petioles of 1.5‒2 cm long with a distinct foveola, leaf blade coriaceous and shiny, male flowers 4‒7 in a fascicle, pedicel 2.5 cm, white petals, stamens 20, in 4 bundles, fused to halfway, alternating with verrucose disc lobes. The sketch at BM shows large elliptic-obovate leaves with slightly acuminate apex and a fasciculate inflorescence with three flowers on 22–28 mm long pedicels.

Again, this description, as well as the sketch, fit *Garcinia smeathmannii* and for now we regard it as a synonym of that species.

## Supplementary Material

XML Treatment for Garcinia quadrifaria chromocarpa

XML Treatment for Garcinia quadrifaria quadrifari

XML Treatment for Garcinia obliqua

XML Treatment for Garcinia gabonensis

XML Treatment for Garcinia arbuscula

XML Treatment for Garcinia danckelmanniana

XML Treatment for Garcinia laurifolia

XML Treatment for Garcinia tschapensis
